# Ribose 5-Phosphate Isomerase B Knockdown Compromises *Trypanosoma brucei* Bloodstream Form Infectivity

**DOI:** 10.1371/journal.pntd.0003430

**Published:** 2015-01-08

**Authors:** Inês Loureiro, Joana Faria, Christine Clayton, Sandra Macedo-Ribeiro, Nuno Santarém, Nilanjan Roy, Anabela Cordeiro-da-Siva, Joana Tavares

**Affiliations:** 1 Parasite Disease Group, Instituto de Biologia Molecular e Celular da Universidade do Porto, Porto, Portugal; 2 Zentrum für Molekulare Biologie der Universität Heidelberg, DKFZ-ZMBH cv Alliance, Heidelberg, Germany; 3 Protein Crystallography Group, Instituto de Biologia Molecular e Celular da Universidade do Porto, Porto, Portugal; 4 Ashok & Rita Patel Institute of Integrated Study & Research in Biotechnology & Allied Sciences, New Vallabh Vidyanagar, Dist-Anand, Gujarat, India; 5 Departamento de Ciências Biológicas, Faculdade de Farmácia da Universidade do Porto, Porto, Portugal; Northeastern University, United States of America

## Abstract

Ribose 5-phosphate isomerase is an enzyme involved in the non-oxidative branch of the pentose phosphate pathway, and catalyzes the inter-conversion of D-ribose 5-phosphate and D-ribulose 5-phosphate. Trypanosomatids, including the agent of African sleeping sickness namely *Trypanosoma brucei*, have a type B ribose-5-phosphate isomerase. This enzyme is absent from humans, which have a structurally unrelated ribose 5-phosphate isomerase type A, and therefore has been proposed as an attractive drug target waiting further characterization. In this study, *Trypanosoma brucei* ribose 5-phosphate isomerase B showed *in vitro* isomerase activity. RNAi against this enzyme reduced parasites' *in vitro* growth, and more importantly, bloodstream forms infectivity. Mice infected with induced RNAi clones exhibited lower parasitaemia and a prolonged survival compared to control mice. Phenotypic reversion was achieved by complementing induced RNAi clones with an ectopic copy of *Trypanosoma cruzi* gene. Our results present the first functional characterization of *Trypanosoma brucei* ribose 5-phosphate isomerase B, and show the relevance of an enzyme belonging to the non-oxidative branch of the pentose phosphate pathway in the context of *Trypanosoma brucei* infection.

## Author Summary

Within the non-oxidative branch of the pentose phosphate pathway, ribose 5-phosphate isomerase catalyzes the inter-conversion of ribose 5-phosphate and ribulose 5-phosphate. There are two types of ribose 5-phosphate isomerase, namely A and B. The presence of type B in *Trypanosoma brucei*, and its absence in humans, make this protein a promising drug target. African sleeping sickness is a serious parasitic disease that relies on limited chemotherapeutic options for control. In our study, a functional characterization of *Trypanosoma brucei* ribose 5-phosphate isomerase B is reported. Biochemical studies confirmed enzyme isomerase activity and its downregulation by RNAi affected mainly parasites infectivity *in vivo*. Overall this study shows that ribose 5-phosphate isomerase depletion is detrimental for parasites infectivity under host pressure.

## Introduction

African sleeping sickness is a vector borne disease of mammals, caused by *Trypanosoma brucei* (*T. brucei*), for which the development of more effective, safe, and affordable chemotherapies remains a major goal. Vaccines are unlikely to be suitable [1]–[3], and therefore disease control relies exclusively on chemotherapy. The glucose-based metabolism is a key metabolic pathway for bloodstream forms, the mammalian infective stages. The absence of a fully functional mitochondrion along with a remarkable high proliferation rate makes parasites entirely dependent on glucose [4], [5]. The glucose-based metabolism comprises two pathways: the glycolytic pathway and the pentose phosphate pathway (PPP). Despite using the same substrate, the pathways have different functions. Glycolysis catabolizes glucose for ATP requirements, while PPP includes an oxidative branch, mainly involved in the maintenance of cell redox homeostasis, and a non-oxidative branch in which ribose 5-phosphate is produced for nucleotide and nucleic acid synthesis. Enzymes involved in the PPP non-oxidative branch include ribose-5-phosphate isomerase, ribulose-5-phosphate epimerase, transaldolase and transketolase, and in contrast with enzymes involved in the glycolysis [6]–[15] or in the oxidative PPP [16], [17], have been less studied. In *T. brucei*, enzymes of the non-oxidative branch downstream ribose-5-phosphate isomerase are apparently developmentally regulated [18]. Ribose 5-phosphate epimerase and transketolase activities were only detected in procyclics, the parasite form present in the insect vector. This suggests that in the mammalian host, bloodstream forms constrain sugar metabolism to the production of ribose-5-phosphate and NADPH via the oxidative phase of the PPP, most likely to meet the remarkably high proliferation rate of these parasites [19], and/or to protect themselves against a variety of reactive oxygen and nitrogen species [20], [21] in a context of an *in vivo* infection.

Ribose-5-phosphate isomerase (Rpi) catalyzes the inter-conversion between ribulose-5-phosphate (Ru5P) and ribose 5-phosphate (R5P). Contrary to trypanosomatids, which have a Rpi type B (RpiB), the presence of a structurally unrelated Rpi type A (RpiA) in humans together with the adverse phenotype observed in *rpiA^-^*/*rpiB^-^* knockout *Escherichia coli* (*E. coli*) [22] have led to suggest RpiB as an attractive drug target candidate that waits further characterization.

In this study, we investigate the importance of RpiB in *T. brucei* bloodstream form viability and infectivity.

## Materials and Methods

### Ethics statement

All experiments were carried out in accordance with the IBMC.INEB Animal Ethics Committees and the Portuguese National Authorities for Animal Health guidelines, according to the statements on the directive 2010/63/EU of the European Parliament and of the Council. IL, JT and ACS have an accreditation for animal research given from Portuguese Veterinary Direction (Ministerial Directive 1005/92).

### Parasite culture

Procyclic and bloodstream *T. brucei* Lister 427 were cultivated in MEM-Pros and HMI-9 medium, respectively, as previously described [23]. Bloodstream forms containing pHD1313 [24] were maintained with 0.2 µg/ml phleomycin.

### Cloning of trypanosomes *RPIB* genes

Ribose 5-phosphate isomerase B genes from *T. brucei* (*TbRPIB*) and *T. cruzi* (*TcRPIB*) were obtained by performing PCR on genomic DNA from *Trypanosama brucei* TREU927 and *Trypanosoma cruzi* CL Brener Non-Esmeraldo-like. Fragments of the open reading frames of *TbRPIB* (Tb927.11.8970; chromosome Tb927_11_v5 from 2,462,183 to 2,463,307) and *TcRPIB* (Tc00.1047053508601.119; chromosome TcChr30-P from 475,724 to 476,203) were PCR-amplified using a Taq DNA polymerase with proofreading activity (Roche). The primers were as follows: sense primer 5′ - CAATTTCCATATGACGCGCAAGGTGGC - 3′ and antisense primer 5′ - CCCAAGCAAGCTTCTAACAACCATTCG - 3′, sense primer 5′ - CAATTTCCATATGACGCGCCGAGTCGC - 3′ and antisense primer 5′ - CCCAAGCGAATTCTCATTTTACCCCTTTG - 3′, respectively. PCR conditions were as follows: initial denaturation (2 min at 94°C), 35 cycles of denaturation (30 s at 94°C), annealing (30 s at 40°C) and elongation (2 min at 68°C) followed by a final extension step (10 min at 68°C); initial denaturation (2 min at 94°C), 35 cycles of denaturation (30 s at 94°C), annealing (30 s at 58°C) elongation (2 min at 68°C) and a final extension step (10 min at 68°C), respectively. The PCR products were isolated from a 1% agarose gel, purified by the Qiaex II protocol (Qiagen), and cloned into a pGEM-T Easy vector (Promega) and sent to Eurofins MWG (Germany) for sequencing. All fragments were checked against the *T. brucei* and *T. cruzi* genome sequence database (http://www.genedb.org) using Blast to ensure their specificity.

### Expression and purification of poly-His-tagged recombinant *Tb*RpiB and *Tc*RpiB

The *TbRPIB* and *TcRPIB* genes were excised from the pGEM-T Easy vector (using NdeI/EcoRI restriction enzyme combination), gel purified and subcloned into pET28a(+) expression vector (Novagen). The resulting constructs presented a poly-His tag (6× Histidine residues) at the N-terminal and were used to transform *E. coli* BL21DE3 cells. Both recombinant proteins were expressed by induction of log-phase cultures (500 ml; OD*_600_*  = 0.6) with 0.5 mM IPTG (isopropyl-β-D- thiogalactopyranoside) for 3 h at 37°C and agitation at 250 rpm/min. Bacteria were harvested by centrifugation (4000 rpm, for 40 min, at 4°C), resuspended in 20 ml of buffer A (0.5 M NaCl, 20 mM Tris.HCl, pH 7.6). The sample was sonicated, according to the following conditions: output 4, duty cycle 50%, 10 cycles with 15 s each. Centrifugation (4000 rpm, for 60 min, at 4°C) was followed to obtain the bacterial crude extract. The recombinant enzymes were purified in one step using Ni^2+^ resin (ProBond) pre-equilibrated in buffer A. The column was washed sequentially with 2–3 ml of the buffer A, 20 ml of the bacterial crude extract, 2 ml of buffer A 25 mM imidazole, 2 ml of buffer A 30 mM imidazole, 2 ml of buffer A 40 mM imidazole, 2 ml of buffer A 40 mM imidazole, 2 ml of buffer A 50 mM imidazole, 10 ml of buffer A 100 mM imidazole, 5 ml of buffer A 500 mM imidazole and 8 ml of buffer B (1 M imidazole, 0.5 M NaCl, 200 mM Tris, pH 7.6). *Tb*RpiB and *Tc*RpiB were eluted in the fractions of buffer A containing between 100 and 500 mM of imidazole. Dialysis was performed against 100 mM Tris/HCl (pH 7.6).

To generate rat polyclonal antibody against *Tb*RpiB, and rabbit polyclonal antibodies against *Tb*RpiB and *Tc*RpiB, each animal was first immunized with 150 µg of recombinant protein. After 2 weeks, 4 boosts with 100 µg of recombinant *Tb*RpiB or *Tc*RpiB were given weekly. The collected blood samples were centrifuged to obtain the sera.

### Protein alignments and homology models

Multiple sequence alignments were performed in ClustalW [25] and images prepared with Aline, Version 011208 [26]. Homology models were obtained in SWISS-MODEL, using PDB accession code 3K7S as a template [27]–[29]. 3D structures were rendered with PyMOL (The PyMOL Molecular Graphics System, Version 1.3, Schrödinger, LLC).

### Enzyme assays

*Tb*RpiB activity was assessed through *Km* determination for R5P and Ru5P, through 4-deoxy-4-phospho-D-erythronohydroxamic acid (4-PEH) (kindly provided by Dr. Laurent Salmon) inhibitory capacity against *Tb*RpiB, and through 4-PEH inhibition mechanism characterization. Firstly, to determine the *Km* for R5P and to characterize 4-PEH-inhibition mechanism, a direct spectrophotometric method at 290 nm [30] was used, to quantify Ru5P formation. *Km* determination was performed at R5P concentrations in a range between 3.1 and 50 mM in Tris/HCl (pH 7.6). For 4-PEH inhibition mechanism characterization, the experiment was performed in the presence of 0.5 µg of enzyme and 0.1, 0.4, 0.7 or 1 mM of inhibitor. All inhibitors were tested in the presence of 3.1 mM R5P. A negative control was made using heat inactivated enzyme. The *Tc*RpiB enzyme was used as a positive control [31]. A calibration curve for Ru5P, using the referred method, was established to determine enzyme activity. An absorbance of 0.0381 at 290 nm was considered for 1 mM Ru5P. To determine the *Km* for Ru5P and to test 4-PEH inhibition as well, a modification of Dische's Cysteine-Carbazole method was used [32]. To determine *Km*, an incubation mixture contained 5 µl of 0.05 µg of enzyme in buffer A [100 mM Tris/HCl (pH 8.4), 1 mM EDTA and 0.5 mM 2-mercaptoethanol] plus 5 µl of Ru5P, giving final concentrations between 0.625 and 10 mM Ru5P, was used. For inhibition assay, Ru5P concentration used was 1.25 mM. Incubation was done for 10 min at room temperature. Following incubation, 15 µl of 0.5% cysteinium chloride, 125 µl of 75% (v/v) sulfuric acid and 5 µl of a 0.1% solution of carbazole in ethanol were added. After 30 min standing at room temperature, the *A_546_* was determined. A blank without enzyme was run for each substrate or inhibitor concentration. Reaction linearity was checked varying enzyme concentration and time. To estimate the remaining Ru5P, a calibration curve was generated. In this assay conditions, 1 mM of Ru5P gave an *A_546_* of 0.270 in a final reaction volume of 155 µl.

### Immunofluorescence

For anti-*Tb*RpiB antibodies validation, cells from log-phase cultures of *T. brucei* RNAi cell lines and wt strain were centrifuged and resuspended at 10^6^/ml in PBS. The cells were fixed in µ-Chamber 12 well (Ibidi) for 15 min, at room temperature, in PBS containing 4% p-formaldehyde, washed twice with PBS, and then permeabilized in PBS containing 0.1% of Triton X-100. The coverslips were incubated in PBS containing 10% FCS during 60 min, at room temperature, in a humidified atmosphere and washed twice with PBS/2% FCS. Then, incubated with primary rat or rabbit polyclonal antibodies against *Tb*RpiB (1∶100 and 1∶1000 respectively, both diluted in blocking solution) overnight, at 4°C, followed by two washes with PBS/2% FCS (5 min each one). Subsequently, cells were incubated with Alexa Fluor 647 conjugated goat anti-rat or Alexa Fluor 488 conjugated goat anti-rabbit secondary antibodies (Molecular probes from Life technologies) (1∶500 diluted in blocking solution) for 1 h at room temperature in an humidified atmosphere, then washed twice with PBS. The coverslips were then stained and mounted with Vectashield-DAPI (Vector Laboratories, Inc.). Images were captured using fluorescence microscope AxioImager Z1 and software Axiovision 4.7 (Carl Zeiss, Germany). Pseudo-coloring of images were carried out using ImageJ software (version 1.43u). In case of *Tb*RpiB immunolocalization, bloodstream form *T. brucei* wt cells were probed using primary rat anti-*Tb*RpiB (1∶100 diluted in blocking solution) and primary rabbit polyclonal antibody against aldolase (glycosome marker, 1∶5000 diluted in blocking solution). Cells were then incubated with biotin conjugated goat anti-rat (1∶500 diluted in blocking solution) (BD Pharmingen) for 1 h room temperature in a humidified atmosphere, then washed twice with PBS/2% FCS. Subsequently, cells were incubated with Alexa Fluor 647 conjugated goat anti-rabbit (Molecular probes, Life technologies) and Streptavidin-FITC (BD Pharmingen) secondary antibodies (1∶1000 diluted in blocking solution) for 1 h at room temperature in an humidified atmosphere, then washed twice with PBS. Vertical stacks were captured, using an confocal microscope Leica TCS SP5II and LAS 2.6 software (Leica Microsystems, Germany). Mean fluorescence intensity of aldolase and RpiB was determined in each stack for the projected co-localization areas. Quantifications were carried out using ImageJ software (version 1.43u).

### Digitonin permeabilization

For each sample condition, bloodstream cells were washed once with cold trypanosome homogenisation buffer (THB), composed by 25 mM Tris, 1 mM EDTA and 10% sucrose, pH = 7.8. Just before cell lyses, leupeptin (final concentration of 2 µg/ml) and different digitonin quantities (final concentrations of 5, 12.5, 25, 50, 100, 150 and 200 ug/ml) were added to 500 µl of cold THB, for cell pellet resuspension. Untreated cells (0 µg/ml of digitonin) and those completely permeabilized (total release, the result of incubation in 0.5% Triton X-100) were used for comparison. Each sample condition was incubated 60 min on ice, and then centrifuged at 2000 rpm, 4°C, for 10 min. Supernatants were taken and 500 µl of cold THB was added to each pellet. All fractions were analysed through Western blot for Rpi (10^8^ cells per well; 1∶1000 polyclonal rabbit anti-*Tb*RpiB as primary antibody), enolase (10^7^ cells per well; 1∶5000 polyclonal rabbit anti-enolase as primary antibody) and aldolase (10^7^ cells per well; 1∶5000 polyclonal rabbit anti-aldolase as primary antibody). HRP-conjugated goat anti-rabbit (1∶5000) was used as secondary antibody.

### Generation of transgenic RNAi cell lines

*TbRPIB* fragment (sense oligo with a BglII – SphI linker 5′ – GAGAAGATCTGCATGCGCGCAAGGTGGCTATCGGTG - 3′, and an antisense oligo with a ClaI – SalI 5′ – GCTAGCTACAGCTGACGGTCCTCCCCGCTGTATG – 3′) was cloned twice in opposite direction on either sides of a “stuffer” of the pHD1144 vector. The resulting construct obtained through HindIII and BglII digestion was cloned into pHD1145. The final construct was transfect into bloodstream forms with pHD1313, and stable individual clones were selected with 7.5 µg/ml of hygromycin. For functional complementation, *TcRPIB* fragment (sense oligo with a HindIII linker 5′ - GAAGCTTATGACGCGCCGAGTCGCAAT - 3′, and an antisense oligo with a BglII linker 5′ - AGATCTTCATTTTACCCCTTTGTTCC - 3′), was cloned in pHD1034 vector (digested with HindIII and BamHI). After transfection [33], individual clones were selected with 0.2 µg/ml of puromycin.

### *In vitro* and *in vivo* analysis of *Tb*RpiB RNAi

For *in vitro* growth curves, cell lines were seeded at 2×10^5^ parasites/ml of complete HMI-9 medium, in the absence and presence of 100 ng/ml of tetracycline (tet). Every 24 h, until day 10, cell growth was monitored microscopically. For *in vivo* infections, after 24 h in the absence of selective drugs, and then a further 48 h of tet induction, 10^4^ wt and transgenic parasites were inoculated intraperitoneally in 6–8 weeks old BALB/c mice (n = 3–8). 48 h prior infection, the RNAi induced mice were treated with 1 mg/ml doxycycline hyclate and 5% sucrose containing water [34], while RNAi non-induced mice were given standard water. Parasitaemia was measured daily from the six day post-infection through tail blood extraction, during a period which all mice in the group were alive.

### Northern blot analysis

Total RNA was isolated from ≈2×10^7^ bloodstream forms using Trizol reagent (Life Technologies). 10 µg RNA were directly separated by overnight formaldehyde agarose-gel electrophoresis, transferred onto a nylon membrane by capillarity and fixed by UV irradiation. The membrane was prehybridized in a hybridization bottle in 5× SSC, 0.5% SDS with salmon sperm DNA (200 µg/ml) and 1× Denhardt's solution for 2 hours at 65°C. *TbRPIB* and signal recognition particle (*SRP*; Tb927.8.2861_7SL) probes were generated by PCR in the presence of [^32^P]-labelled dCTP using Prime-It RmT random primer labelling kit (Stratagene) followed by purification using QIAquick Nucleotide Removal Kit (QIAGEN). Denaturated radioactive probes were added to the prehybridization solution at 65°C and incubated overnight. After rinsing the membrane twice for 5 min. with 2× SSC/0.1% SDS, the probes were washed out with two washes of 30 minutes in 0.1× SSC/0.1% SDS at 65°C and the membrane exposed on a Fugifilm FLA-3000 reader screen. ImageJ software (version 1.43u) was used for RNA quantification.

### Protein extracts and western blot analysis

Cell free extracts were obtained in RIPA buffer (20 mM Tris-HCl (pH 7.5), 150 mM NaCl, 1 mM Na_2_EDTA, 1 mM EGTA, 1% NP-40, 1% sodium deoxycholate, 2.5 mM sodium pyrophosphate, 1 mM b-glycerophosphate, 1 mM Na_3_VO_4_), with freshly-added complete protease inhibitor cocktail (Roche Applied Science). The total protein amount was quantified using Biorad Commercial Kit (Reagents A, B and S) and the samples were then kept at -80°C. For analysis of parasites collected from mice, trypanosomes were purified from mouse blood using a DE-52 (Whatman) column [35].

For Western blotting, 10 µg of recombinant *Tb*RpiB and *Tc*RpiB proteins were resolved in 15% SDS/PAGE (Tris-Tricine gel), while 30 µg of total soluble cell extract and 10^7^ parasites were resolved in 12% Tris-Glycine SDS/PAGE, and all were then transferred on to a nitrocellulose Hy-bond ECL membrane (Amersham Biosciences). The membrane was blocked in 5% (w/v) non-fat dried skimmed milk in PBS/0.1% Tween-20 (blocking solution), followed by incubation with an anti-His-tag rabbit antibody (MicroMol-413) (1∶1000) or a combination of an anti-*Tb*RpiB rabbit antibody (1∶1000) with an anti-aldolase rabbit antibody (1∶5000) in blocking solution at 4°C overnight, respectively. Blots were washed with PBS/0.1% Tween-20 (3 times 15 min). Horseradish peroxidase-conjugated goat anti-rabbit IgG (Amersham) (1∶5000 for 1 h, at room temperature) was used as the secondary antibody. The membranes were developed using SuperSignal WestPico Chemiluminescent Substrate (Pierce). ImageJ software (version 1.43u) was used for protein bands semi-quantification.

### Statistical analysis

Student's t-test and Graphpad Prism Software (version 5.0) were used. *p* values ≤0.05 were considered to be statistically significant (* *p*≤0.05, ** *p*≤0.01, *** *p*≤0.001).

## Results

### *Tb*RpiB biochemical properties

An open reading frame with sequence similarity to RpiB was identified both in *T. brucei* (Tb927.11.8970) and in *T. cruzi* (Tc00.1047053508601.119) genomes. Protein sequence alignment using ClustalW [25] revealed 67% identity for *Tb*RpiB *versus Tc*RpiB, and both proteins show no similarity with human ribose 5–phosphate isomerase A. *Tc*RpiB and *Tb*RpiB contain 159 and 155 amino acids residues per monomer, respectively. Protein multiple sequence alignment of RpiB from *T. cruzi* CL Brener Esmeraldo-like (Tc00.1047053509199.24; PDB accession code 3K7S [36]), *T. cruzi* CL Brener Non-Esmeraldo-like (Tc00.1047053508601.119) and *T. brucei* (Tb927.11.8970) is shown in S1A Fig. The scale colour, from cyan (low-similarity residues) to red (high-similarity residues), underlines the degree of similarity between the three protein sequences, also seen in the *Tc*RpiB (Esmeraldo like strain) ribbon representation (S1B Fig.). The superposition of *Tc*RpiB (Esmeraldo like strain) structure (grey) (PDB code 3K7S), with the homology models generated for *Tc*RpiB (Non Esmeraldo like strain) (purple) and *Tb*RpiB (blue) show a high structural homology and strict conservation of the residues involved in R5P binding pocket (S1A, C Fig.).

**Figure 1:**
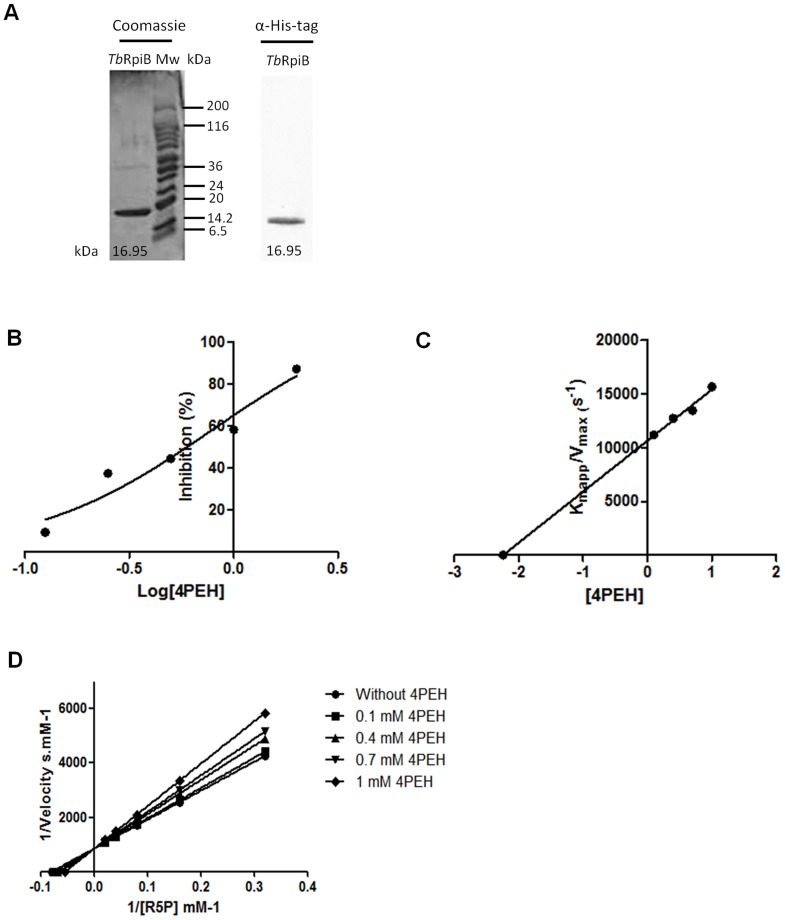
Biochemical properties of *Tb*RpiB expressed in *E. coli*. (A) 10 µg of *Tb*RpiB recombinant protein analyzed by SDS-PAGE and Coomassie blue staining. Mw, molecular weight marker. Western blot analysis of his-tagged recombinant protein probed with rabbit anti-histidine monoclonal antibody (MicroMol-413) (1∶1000). (B) Inhibition (%) of *Tb*RpiB activity by 4PEH. (C) Plot of *K*_mapp_/*V*_max_ *versus* 4PEH concentrations; *K*_i_ corresponds to the symmetric value of the X-axis intersection. (D) Plots showing the effect of different 4PEH concentrations on the inverse of the initial velocity *versus* the inverse of several concentrations of R5P. (B–D) The values correspond to the means ± standard deviation of two replicates, and data is representative of three independent experiments.

Biochemical studies were performed using histidine-tagged fusion *Tb*RpiB and *Tc*RpiB (positive control) proteins expressed in *E. coli* and purified under non-denaturing conditions (Figs. 1A, S2A). The *T. brucei* and *T. cruzi* [31] enzymes have *in vitro* ribose 5-phosphate isomerase activity, as these proteins can use both R5P and Ru5P as substrates. For R5P, *T. brucei* protein showed a significantly higher *K*_m_ (2.8 fold increase, *p*<0.05), but not a lower maximum velocity (*V*_max_) or catalytic constant (*k*_cat_) compared to *T. cruzi* enzyme (Table 1 and S2B Fig.). For Ru5P, the *K_m_* of the *T. brucei* protein was not significantly different from that of the *T. cruzi* enzyme value, but the *V*_max_ and *k*_cat_ were higher (≈1.5 fold, *p*<0.05) (Table 1 and S2B Fig.). Both the *T. brucei* and the *T. cruzi* enzymes exhibited significant lower *K*_m_s for Ru5P than for R5P, (5.2 fold, *p*<0.05 and 3.7 fold, *p*<0.01, respectively), suggesting the reaction occurs preferentially from Ru5P to R5P. The turnover values (*k*_cat_) were found to be significantly higher for Ru5P than for R5P, in both *T. brucei* (*p* = 0.001) and *T. cruzi* (*p*<0.001) enzymes (Table 1 and S2B Fig.).

**Table 1:** *Tb*RpiB kinetic parameters.

	R5P to Ru5P	Ru5P to R5P
K_m_ (mM)	12.50±4.43	2.39±0.94
V_max_ ×10^−3^(mM.s^−1^)	1.17±0.16	5.84±0.79
k_cat_ (s^−1^)	12.00±1.58	39.44±5.32
k_cat_/K_m_ (M^−1^.s^−1^)	9.60×10^2^	1.64×10^4^

The values are the means ± standard deviation obtained from 3 independent experiments.

The reaction mechanism of ribose 5-phosphate isomerase involves two steps: an initial opening of the furanose ring of R5P, followed by the aldolase-ketose isomerisation, via a cis-enediolate high energy intermediate [31]. 4-PEH has been described to act as a competitive inhibitor which compromises the binding of 1,2-*cis*-enediolate intermediate [37]. The inhibitory capability of 4-PEH was screened *in vitro*, resulting in an IC50 of 0.8 mM and 0.7 mM for *Tb*RpiB (Fig. 1B) and *Tc*RpiB (S2C Fig.), respectively, with *K*_i_ values of 2.2 (Fig. 1C) and 1.6 mM (S2D Fig.). 4-PEH showed, as expected, a competitive inhibition behaviour, once using increasing concentrations of inhibitor, a progressive increase in the *K*_m_ for R5P without *V*_max_ alteration was observed (Figs. 1D, S2E). The inhibitor behaviour, and also the IC_50_ and the *K_i_* values are in agreement to what was described before for *T. cruzi* enzyme [31], [36]. 4-PEH was also reported as a potent inhibitor against *Mycobacterium tuberculosis* RpiB [37].

Undoubtedly, *Tb*RpiB has isomerase activity and uses preferentially ribulose 5-phosphate as a substrate.

### *Tb*RpiB expression and subcellular localization

**Figure 2:**
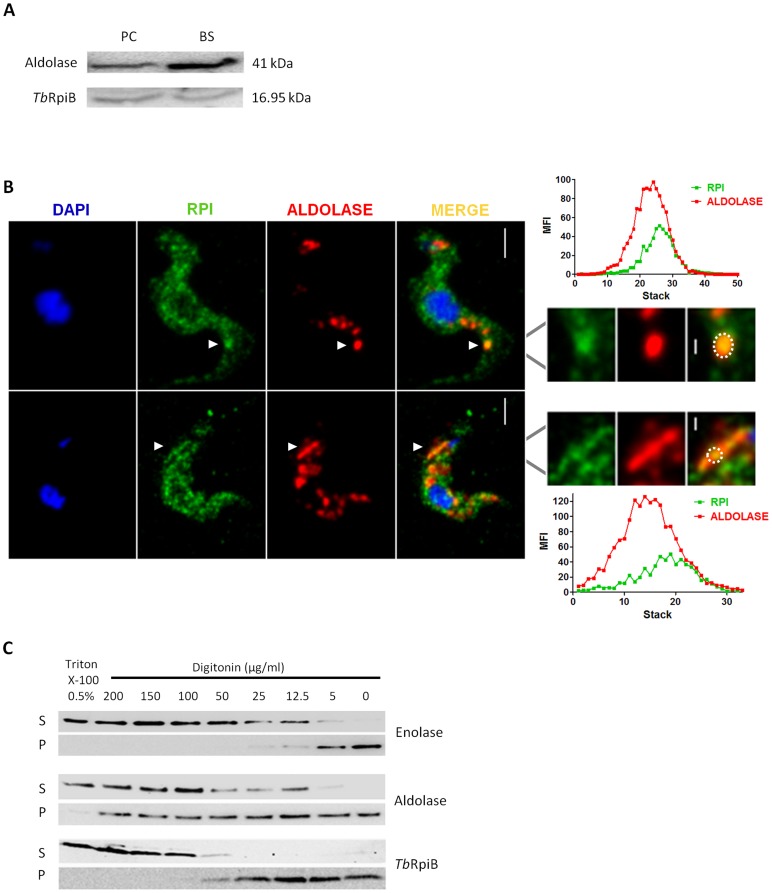
*Tb*RpiB expression within life cycle stages and localization in bloodstream forms. (A) RpiB expression in *T. brucei* life-cycle stages; 30 µg of protein from bloodstream (BS) and procyclic (PC) total lysates was analysed by Western blot probed with rabbit anti-*Tb*RpiB (1∶1000) and anti-aldolase (loading control; 1∶5000) polyclonal antibodies. Data is representative of three independent experiments. (B) Immunofluorescence analysis by confocal microscopy of bloodstream forms *Tb*RpiB. Nuclear and kinetoplast DNA labelled by DAPI staining (blue). RpiB (green) and aldolase (red) were labelled respectively with rat anti-*Tb*RpiB (1∶100) and rabbit anti-aldolase (1∶5000) antibodies. White arrowheads indicate RpiB and aldolase co-localization areas that are magnified in the right panels. Mean fluorescence intensity (MFI) of aldolase (red) and RpiB (green) in these co-localization areas (white dotted circle) were determine for each stack. Images are maximal Z-projections of 50 and 33 contiguous stacks separated by 0.1 µm. Scale Bars: 2.5 (top left panel), 5 (below left panel), 0.5 (top right panel) and 1 µm (below right panel). (C) Supernatant (S) and pellet (P) fractions obtained with different concentrations of digitonin were subjected to Western blot analysis and probed with rabbit antibodies against *Tb*RpiB (1∶1000), enolase (cytoplasmic marker; 1∶5000), and aldolase (glycosome marker; 1∶5000). Data is representative of two independent experiments. Untreated cells and those completely permeabilized by incubation with 0.5% Triton X-100 [total release (TR)] were used as controls.

Rabbit and rat polyclonal antibodies were generated against the *Tb*RpiB recombinant protein. Antibody specificity was validated, as induction of RpiB RNAi resulted in a decrease in the fluorescence intensity of bloodstreams when compared to non-induced parasites (S3A, B, C Fig.). Similarly a significant decrease on RpiB levels in the extracts of *Tb*RpiB RNAi induced parasites is shown by Western blot. Rat and rabbit antibodies specificity against RpiB can be appreciated on the whole Western blot membranes (S3D, E Fig.). Using rabbit polyclonal antibody against parasite extracts, *Tb*RpiB was found more abundant in procyclic forms than in bloodstream forms (Fig. 2A). To ascertain RpiB subcellular localization in bloodstream forms, two complementary approaches, immunofluorescence and digitonin fractionation, were performed. Fluorescent confocal microscopy analysis suggests that *Tb*RpiB despite being localized mainly in the cytosol can be also found in glycosomes due to colocalization with the glycosomal marker, aldolase [38] (Fig. 2B). Upon digitonin fractionation, RpiB showed an intermediate pattern between the glycosomal marker, aldolase (still partially in the pellet after 200 µg/ml digitonin treatment) and the cytosolic marker, enolase (almost all in supernatant with 25 µg/ml digitonin), being practically released with 100 µg/ml digitonin (Fig. 2C). In conclusion, RpiB localizes mainly in the cytosol of bloodstream forms.

### *In vitro* and *in vivo* analysis of *Tb*RpiB RNAi

**Figure 3:**
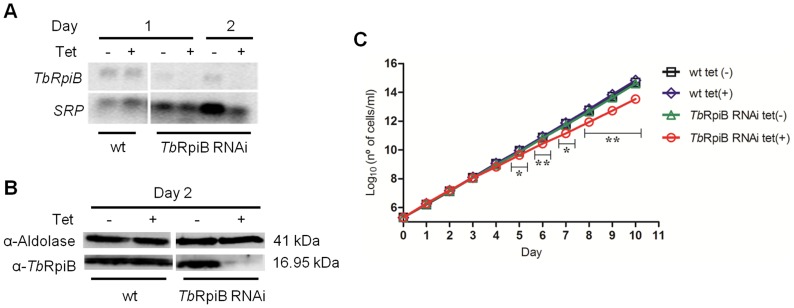
*In vitro* effect of RNAi-mediated RpiB downregulation on *T. brucei* bloodstream forms. (A) Northern and (B) Western blot analysis of mRNA and protein levels, respectively, upon RpiB RNAi. *SRP* and aldolase used as loading controls, respectively. Rabbit anti-*Tb*RpiB (1∶1000) and anti-aldolase (1∶5000) polyclonal antibodies were used as primary antibodies. (C) Growth curve of a wt *versus* a representative RpiB RNAi cell line. Black squares and blue diamonds represent wt growth in the absence or presence of tetracycline (tet) while green triangles and red circles represent RpiB RNAi clone growth in the absence or presence of tet, respectively. Cumulative cell numbers (product of cell number and total dilution) are plotted. Values represent averages from three independent experiments using one representative RpiB RNAi clone and error bars indicate standard deviation. Statistical differences between non-induced and induced *Tb*RpiB RNAi clone are depicted (* *p*≤0.05, ** *p*≤0.01).

To assess if *Tb*RpiB targeting affects *in vitro* bloodstream forms growth, RNAi against RpiB was induced. This resulted in a lower mRNA and protein levels 1 and 2 days post-induction (Fig. 3A and B, respectively). Using ImageJ software we estimate a decrease of approximately 93% of protein levels at 48 h RNAi post-induction. The growth of *Tb*RpiB RNAi tet(-) and wt tet(-) cell lines was shown to be similar (Fig. 3C). A significant decrease of *in vitro* cell proliferation of induced *versus* non-induced RNAi cell lines was seen only after day 4 of the cumulative growth curve (Fig. 3C).

**Figure 4:**
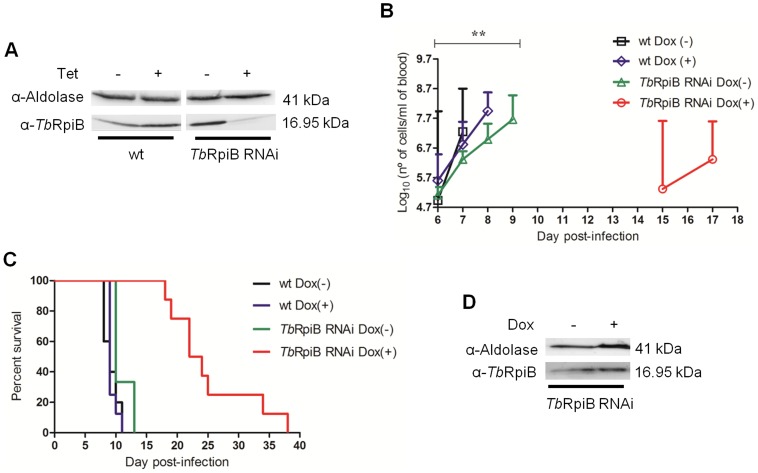
*In vivo* effect of RNAi-mediated RpiB downregulation on *T. brucei* bloodstream forms. (A) Western blot analysis of Rpi protein levels in bloodstream forms 48 h after tet induction, which were used for mice infections. (B) Groups of mice (n = 3–7) were infected intraperitoneally with 10^4^ control wt (black squares and blue diamonds) or a representative RNAi clone (green triangles and red circles). The mice were either untreated (black squares and green triangles) or treated with 1 mg/ml Dox (blue diamonds and red circles) in the water supply. Parasitaemias of each group are shown for the period of time in which there is no mice death. Values are means and errors bars indicate + standard deviation. 5×10^4^ trypanosomes/ml of blood is the detection limit. Mice were culled when parasitaemia reached 10^8^ cells/ml. (C) Kaplan–Meier survival analysis of mice infected with non-induced and induced wt cell line (black and blue line, respectively) *versus* a non-induced and induced representative RNAi clone (green and red line, respectively). Parasitaemias and survival curve are representative of two independent experiments using two different RNAi clones. (D) Western blot analysis of RpiB levels in a representative non-induced and Dox-induced RNAi clone collected from mice before being euthanized confirmed the appearance of RNAi revertants. Statistical differences between non-induced and induced *Tb*RpiB RNAi clone are depicted (** *p*≤0.01).

To test the importance of RpiB for parasite infectivity in a disease model, two groups of BALB/c mice were inoculated with the wt parental cell line and other two groups with the RNAi cell line. Some mice were fed with water containing doxycycline (Dox) to induce downregulation of *Tb*RpiB, whilst the remaining mice were kept as non-induced controls. A Western blot confirmed the reduction of the protein level in 48 h RNAi induced parasites used for mice infections (Fig. 4A). Blood samples were taken from all mice at daily intervals to chart parasitaemia (Fig. 4B). Animals achieving a parasitaemia greater than 10^8^ trypanosomes per millilitre were euthanized. *In vivo* growth of the *Tb*RpiB RNAi Dox(-) trypanosomes was not significantly different from that of wt Dox(-) parasites. However a significant decrease in the parasitaemia of induced *versus* non-induced RNAi cell lines was seen. Within 6 days of inoculation, contrary to mice infected with induced RNAi cell line (in which overall parasitaemias remained below the detection limit, 5×10^4^ trypanosomes/ml), mice infected with control parasites developed high levels of parasitaemia. As a consequence, and in contrast to mice infected with wt and *Tb*RpiB RNAi Dox(-) parasites, which were culled sooner (between eighth to thirteenth day post-infection), *Tb*RpiB RNAi Dox(+) were euthanized from the eighteenth day post-infection (Fig. 4C). Eventually parasitaemia also increased in the *Tb*RpiB RNAi Dox(+) mice, due to the emergence of “RNAi revertants” (Fig. 4D) [39]–[42]. In this way, ribose 5-phosphate isomerase B despite being dispensable *in vitro*, confers optimal *in vitro* growth and is highly relevant for mice infections.

### Complementation of *Tb*RpiB RNAi phenotype

**Figure 5:**
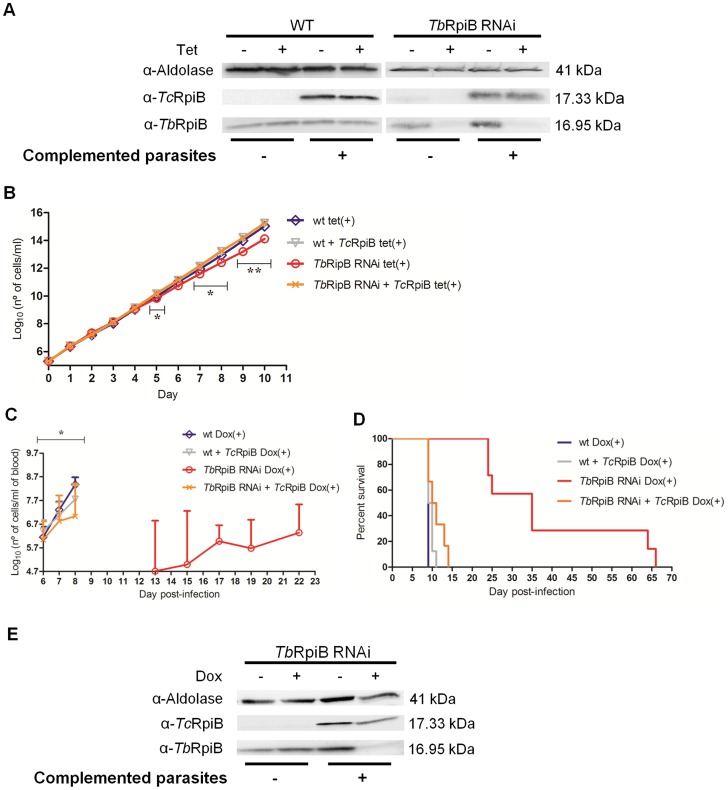
Rescue of RpiB RNAi mediated defect by expression of *Tc*RpiB. (A) Western blot analysis of *Tb*RpiB and *Tc*RpiB levels in bloodstream forms 48 h after tetracycline (tet) induction reveal a decrease of RpiB in non-complemented and complemented *Tb*RpiB RNAi cells, contrary to wt controls. Parasite extracts were probed sequentially, with rabbit anti-*Tb*RpiB (1∶1000) and anti-aldolase (loading control; 1∶5000), and with anti-*Tc*RpiB (1∶1000) primary antibodies. (B) *In vitro* cumulative growth of induced non-complemented and complemented wt bloodstream forms (blue diamond and grey down triangle, respectively) *versus* an induced non-complemented and complemented representative *Tb*RpiB RNAi clone (red circle and orange cross, respectively). Values represent an average of parasite numbers ± standard deviation of two independent experiments from a representative RNAi clone. (C) Groups of mice (n = 6–8) were infected intraperitoneally with 1×10^4^ RNAi induced non-complemented and complemented wt parental cell line (blue diamond and grey down triangle, respectively) *versus* non-complemented and complemented representative *Tb*RpiB RNAi clone (red circle and orange cross, respectively). Mice were treated with 1 mg/ml Dox in the water supply. Mice were culled when parasitaemia reached 10^8^ cells/ml. The mean value of the parasitaemias for each group of mice + standard deviation is shown. (D) Kaplan–Meier survival analysis of mice infected with Dox induced non-complemented and complemented wt cell line (blue and grey lines, respectively) *versus* induced non-complemented and complemented representative *Tb*RpiB RNAi clone (red and orange lines, respectively). Data are representative of two independent experiments of two different RNAi clones. (E) Western blot analysis of RpiB levels in a representative non-complemented and complemented *Tb*RpiB RNAi clone isolated from mice blood before being euthanized, showing the emergence of RNAi revertants only in induced non-complemented RNAi clones. Statistical differences between non-complemented and complemented induced *Tb*RpiB RNAi clone are depicted (* *p*≤0.05, ** *p*≤0.01).

Functional complementation of *T. brucei* RNAi cell lines with the *T. cruzi* homologue was performed, since *Tc*RpiB has *in vitro* isomerase activity and *TcRPIB* nucleotide sequence is sufficiently different to avoid *Tb*RpiB RNAi. Western blot analysis confirmed *Tb*RpiB downregulation only in induced RNAi parasites, and *Tc*RpiB expression exclusively in complemented parasites (Fig. 5A). Cells with RNAi and complemented with *Tc*RpiB grew equally *in vitro* (Fig. 5B), and were almost as virulent *in vivo* (Fig. 5C, D), as the wild-type. RNAi revertants appeared during the course of infection in induced *Tb*RpiB RNAi infected mice, but not in induced complemented *Tb*RpiB RNAi infected mice (Fig. 5E). As a result, complementation restored *in vitro* and *in vivo* phenotypes.

## Discussion

In this study we demonstrated that *Tb*RpiB, like the related *Tc*RpiB and *Leishmania donovani* RpiB (*Ld*RpiB) enzymes, has *in vitro* ribose 5-phosphate isomerase activity [31], [43]. Based on the theoretical homology model, *Tb*RpiB is predicted to be dimeric. Although the dimer comprises a complete functional unit, tetramers are observed in all available RpiB structures except that of *Mycobacterium tuberculosis* RpiB [36]. Similarly to *T. cruzi*, *Clostridium thermocellum* and *Pisum sativum* Rpi enzymes, *Tb*RpiB has the ability of using both R5P or Ru5P as substrates, but with remarkable preference for Ru5P [31], [44], [45]. However, the differences in affinity are more pronounced in trypanosomes enzymes. Indeed, these differences were higher for *Tb*RpiB compared to *Tc*RpiB. Analysis of the three enzymes from trypanosomatids (*Tc*RpiB, *Ld*RpiB and *Tb*RpiB) shows that *Tb*RpiB and *Ld*RpiB have the highest *K*_m_ and *k*_cat_ value for R5P substrate, respectively [31], [43]. Nevertheless, we can speculate that such differences may result in part by the fact that parasite enzymes were expressed and purified as recombinant proteins in bacteria and not purified directly from trypanosomes extracts. Consequently, differences in protein post-transcriptional processing and/or changes in protein conformation cannot be excluded.

RpiB is expressed on *T. brucei* procyclic and bloodstream forms, and our data indicate its higher expression in procyclics. Interestingly, a previous study has shown higher levels of *TbRPIB* mRNA (Tb927.11.8970) in logarithmic phase procyclic forms compared to bloodstream forms [46]. However, its biological meaning, if any, remains to be elucidated.

Regarding RpiB subcellular localization in bloodstream forms, the protein despite found mainly in the cytosol is also present in glycosomes. This might explain why a previous proteomic analysis failed to find *Tb*RpiB enzyme in purified glycosomes [47]. The glycosomal localization observed within the dual-localization can be justified by the presence of a peroxisomal targeting signal, PTS2 (-KVAIGADHI-), at the N-terminus [48]. Moreover, other enzymes of the hexose-monophosphate pathway, although present in glycosomes, were also found mainly within the cytosol (e.g. glucose-6-phosphate dehydrogenase, 6-phosphogluconolactonase and transketolase) [49], [50].

*Tb*RpiB is clearly needed for optimal *in vitro* parasite growth, although we do not know whether it is essential for survival since some protein remained after RNAi. Nevertheless, our results show that *Tb*RpiB is important for parasites infectivity *in vivo*, through the appearance of RNAi revertants and reversion of the phenotype in complemented parasites. Infectivity defects of bloodstreams with reduced levels of *Tb*RpiB were shown on a monomorphic *T. brucei* strain. This strain is abnormally virulent and typically mice do not survive longer than ≈10 days. In the future, it would be interesting to test the role of RpiB in a more chronic infection, as the one caused by pleomorphic strains. Interfering with the PPP non-oxidative branch showed to be detrimental under host pressure, in these highly proliferative parasitic forms, which can be due to a defective production of ribose 5-phosphate towards nucleotide and nucleic acid synthesis. Moreover, another enzyme capable of producing ribose 5-phosphate, ribokinase, is essential for parasites survival since attempts to remove the two alleles were unsuccessful [51].

*Tb*RpiB is not the first protein reported as dispensable under standard laboratory culture conditions but crucial for parasites growth in the animal host [52], [53]. In rich culture conditions, parasites may uptake essential nutrients from the extracellular medium, which may not be as available in blood. Moreover, *in vivo*, parasites need to deal with pressure from the host immune response.

As for other proteins [54], [55], our *in vitro* results differ from the ones achieved in RNA interference target sequencing (RITseq) screen [56]. Indeed, proteins described to be significantly important for parasites fitness by Alsford and colleagues [56] were not in others studies [54], [55]. Despite large-scale RNAi screens have already proved useful, caution should be taken due to some level of false negatives and positives, inherent to high-throughput approaches and more importantly due to off-target effects [57]. Furthermore, variations between different large-scale RNAi screenings were already been reported and explained by the use of different *T. brucei* strains, RNAi constructs and methods for assessing cell growth highlighting the importance of using complementary approaches in such studies [58]. Despite all, both studies are in agreement and show a role for *Tb*RpiB on parasites growth.

To further investigate if bloodstream forms deleted of RpiB are completely cleared in mice, studies with gene knockout parasites should be done.

Overall our results clearly show a role of RpiB for bloodstream *in vitro* optimal growth and more importantly *in vivo* infectivity, but also suggest a conserved role among different *Trypanosoma* species. In conclusion *Tb*RpiB emerges as a new potential therapeutic target against African sleeping sickness.

## Supporting Information

S1 Fig **Sequence alignment and ribbon representation of RpiB protein from trypanosomes.** (A) ClustalW alignment of RpiB from *T. cruzi* CL Brener Esmeraldo-like (Tc00.1047053509199.24; PDB accession code 3K7S), *T. cruzi* CL Brener Non-Esmeraldo-like (Tc00.1047053508601.119) and *T. brucei* (Tb927.11.8970). The residues are colored according to ALSCRIPT Calcons (Aline version 011208) using a predefined colour scheme (red: identical residues; orange to blue: scale of conservation of amino acid properties; white: dissimilar residues). Secondary structure of *Tc*RpiB crystallographic model (PDB code 3K7S) (grey) and the theoretical homology models *Tc*RpiB (Tc00.1047053508601.119) (purple) and *Tb*RpiB (Tb927.11.8970) (blue) are depicted above the alignment. Black circles indicate R5P binding residues. (B) Ribbon representation of *Tc*RpiB Esmeraldo-like (PDB code 3K7S) colored according to the sequence similarity with *Tc*RpiB Non-Esmeraldo-like and *Tb*RpiB as shown in (A). (C) Superposition of *Tc*RpiB structure (PDB code 3K7S) (grey) with *Tc*RpiB (Tc00.1047053508601.119) (purple) and *Tb*RpiB (Tb927.11.8970) (blue) homology models. Ligand color scheme: R5P is shown in yellow (oxygen, pink; phosphorous orange).
(TIF)

S2 Fig **Biochemical properties of** ***Tc*** **RpiB (Tc00.1047053508601.119) expressed in** ***E. coli*** **.** (A) 10 µg of *Tc*RpiB recombinant protein analyzed by SDS-PAGE and Coomassie blue staining. Mw, molecular weight marker. Western blot analysis of his-tagged recombinant protein probed with rabbit anti-histidine monoclonal antibody (MicroMol-413) (1∶1000). (B) Kinetic parameters of direct (R5P to Ru5P) and inverse (Ru5P to R5P) reaction. The values are the means ± standard deviation obtained from 3 independent experiments. (C) Inhibition (%) of *Tc*RpiB activity by 4PEH. (D) Plot of *K*_mapp_/*V*_max_ *versus* 4PEH concentrations; *K*_i_ corresponds to the symmetric value of the X-axis intersection. (E) Plot showing the effect of different 4PEH concentrations on the inverse of the initial velocity *versus* the inverse of several concentrations of R5P. (C–E) The values correspond to the means ± standard deviation of two replicates, and data is representative of three independent experiments.
(TIF)

S3 Fig **Validation of antibodies against** ***Tb*** **RpiB.** Immunofluorescence analysis of *T. brucei* wt or a representative Rpi RNAi clone in the presence or absence of tetracycline (tet). RNAi induced and uninduced cells were grown for 48 h, then fixed and probed with rat polyclonal anti-*Tb*RpiB (A) or rabbit polyclonal anti-*Tb*RpiB (B) antibody and co-stained with DAPI. Bars, 5 µm. (C) Quantification of *Tb*RpiB fluorescence levels in induced cells [Rpi RNAi tet(+), *n* = 30] and uninduced cells [Rpi RNAi tet(−), *n* = 30], using the rat and the rabbit polyclonal anti-*Tb*RpiB antibodies. Data representative of two independent experiments using two different clones. ImageJ software (version 1.43u) was used for fluorescence quantification. *p* value was calculated by Student's t test (*** *p*≤0.001, for both *p*<0.001). (D, E) Whole membrane resulting from Western blot analysis of RpiB levels, in *T. brucei* wt or a representative Rpi RNAi clone, in the presence or absence of tet. The membrane was probed with rat anti-*Tb*RpiB (1∶100) (D) or rabbit anti-*Tb*RpiB (1∶1000) (E), and after membrane stripping, with rabbit anti-aldolase (1∶5000) for loading control.
(TIF)
