# Supplementary figures and images for: Ribose 5-Phosphate Isomerase B Knockdown Compromises Trypanosoma brucei Bloodstream Form Infectivity
Source: PLoS Negl Trop Dis. 2015 Jan 8;9(1):e3430. doi: 10.1371/journal.pntd.0003430 (PMC4287489; doi:10.1371/journal.pntd.0003430)

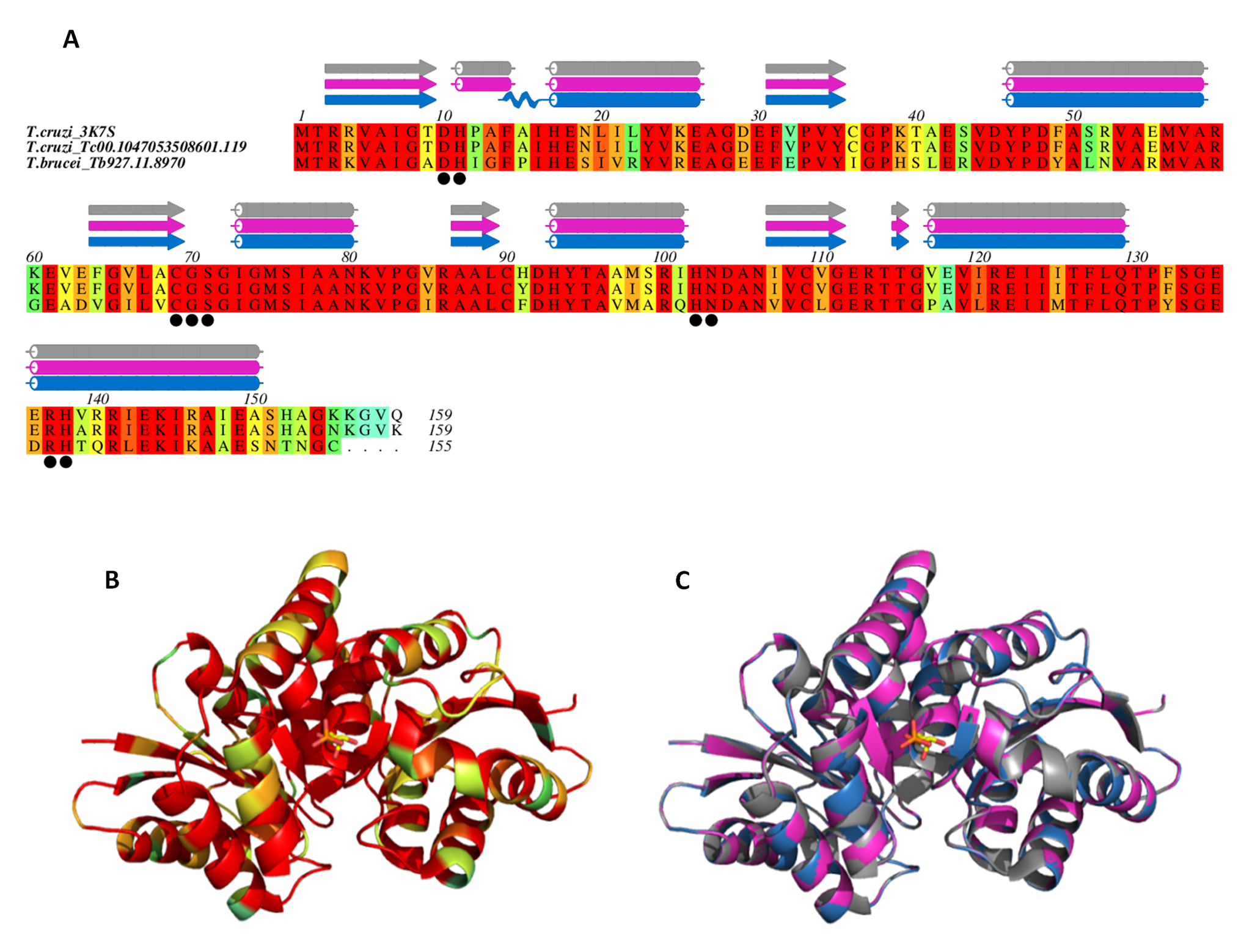

Supplement: S1 Fig — Sequence alignment and ribbon representation of RpiB protein from trypanosomes. (A) ClustalW alignment of RpiB from T. cruzi CL Brener Esmeraldo-like (Tc00.1047053509199.24; PDB accession code 3K7S), T. cruzi CL Brener Non-Esmeraldo-like (Tc00.1047053508601.119) and T. brucei (Tb927.11.8970). The residues are colored according to ALSCRIPT Calcons (Aline version 011208) using a predefined colour scheme (red: identical residues; orange to blue: scale of conservation of amino acid properties; white: dissimilar residues). Secondary structure of TcRpiB crystallographic model (PDB code 3K7S) (grey) and the theoretical homology models TcRpiB (Tc00.1047053508601.119) (purple) and TbRpiB (Tb927.11.8970) (blue) are depicted above the alignment. Black circles indicate R5P binding residues. (B) Ribbon representation of TcRpiB Esmeraldo-like (PDB code 3K7S) colored according to the sequence similarity with TcRpiB Non-Esmeraldo-like and TbRpiB as shown in (A). (C) Superposition of TcRpiB structure (PDB code 3K7S) (grey) with TcRpiB (Tc00.1047053508601.119) (purple) and TbRpiB (Tb927.11.8970) (blue) homology models. Ligand color scheme: R5P is shown in yellow (oxygen, pink; phosphorous orange). (TIF) [file pntd.0003430.s001.tif]

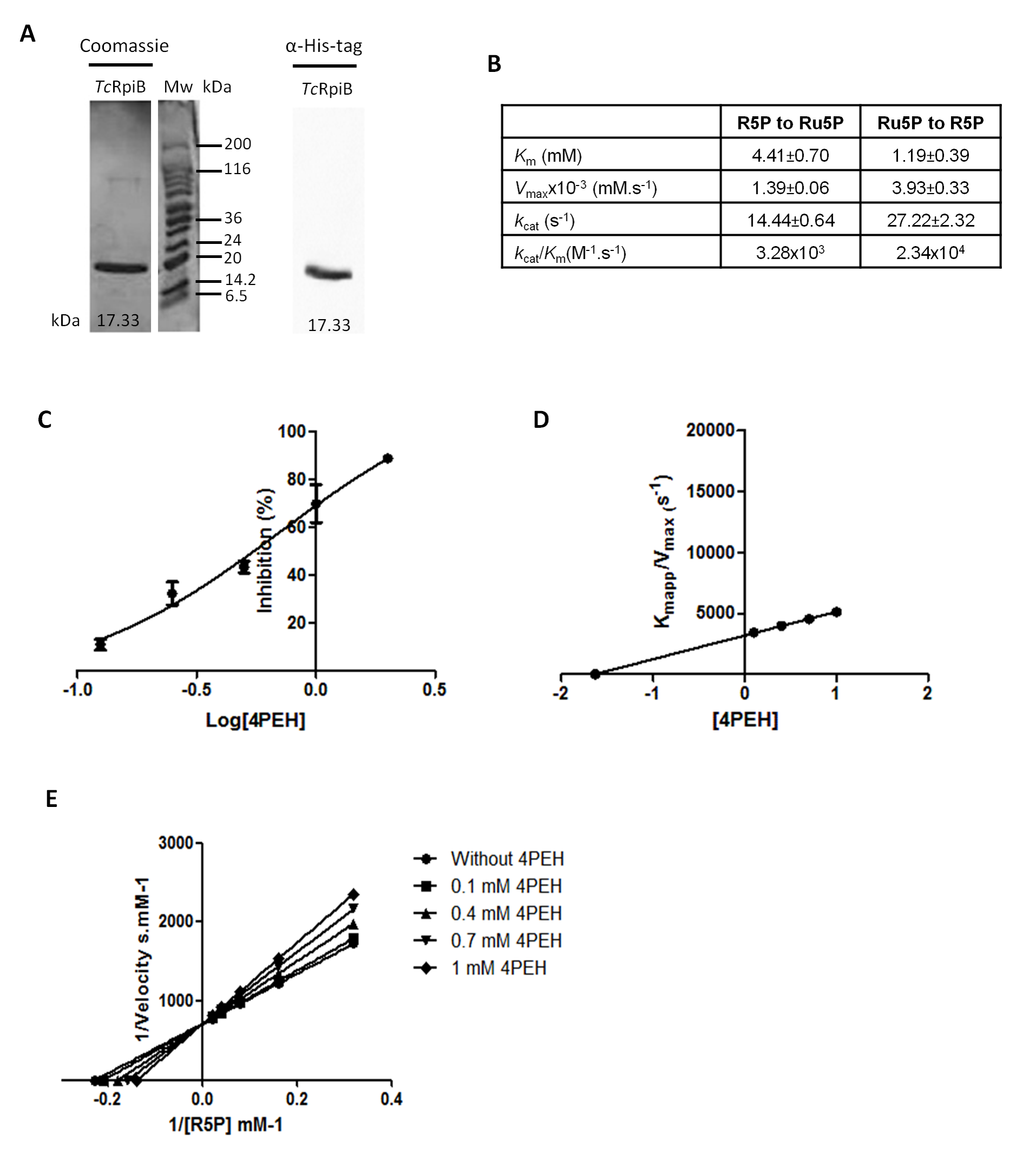

Supplement: S2 Fig — Biochemical properties of Tc RpiB (Tc00.1047053508601.119) expressed in E. coli . (A) 10 µg of TcRpiB recombinant protein analyzed by SDS-PAGE and Coomassie blue staining. Mw, molecular weight marker. Western blot analysis of his-tagged recombinant protein probed with rabbit anti-histidine monoclonal antibody (MicroMol-413) (1∶1000). (B) Kinetic parameters of direct (R5P to Ru5P) and inverse (Ru5P to R5P) reaction. The values are the means ± standard deviation obtained from 3 independent experiments. (C) Inhibition (%) of TcRpiB activity by 4PEH. (D) Plot of K mapp/V max versus 4PEH concentrations; K i corresponds to the symmetric value of the X-axis intersection. (E) Plot showing the effect of different 4PEH concentrations on the inverse of the initial velocity versus the inverse of several concentrations of R5P. (C–E) The values correspond to the means ± standard deviation of two replicates, and data is representative of three independent experiments. (TIF) [file pntd.0003430.s002.tif]

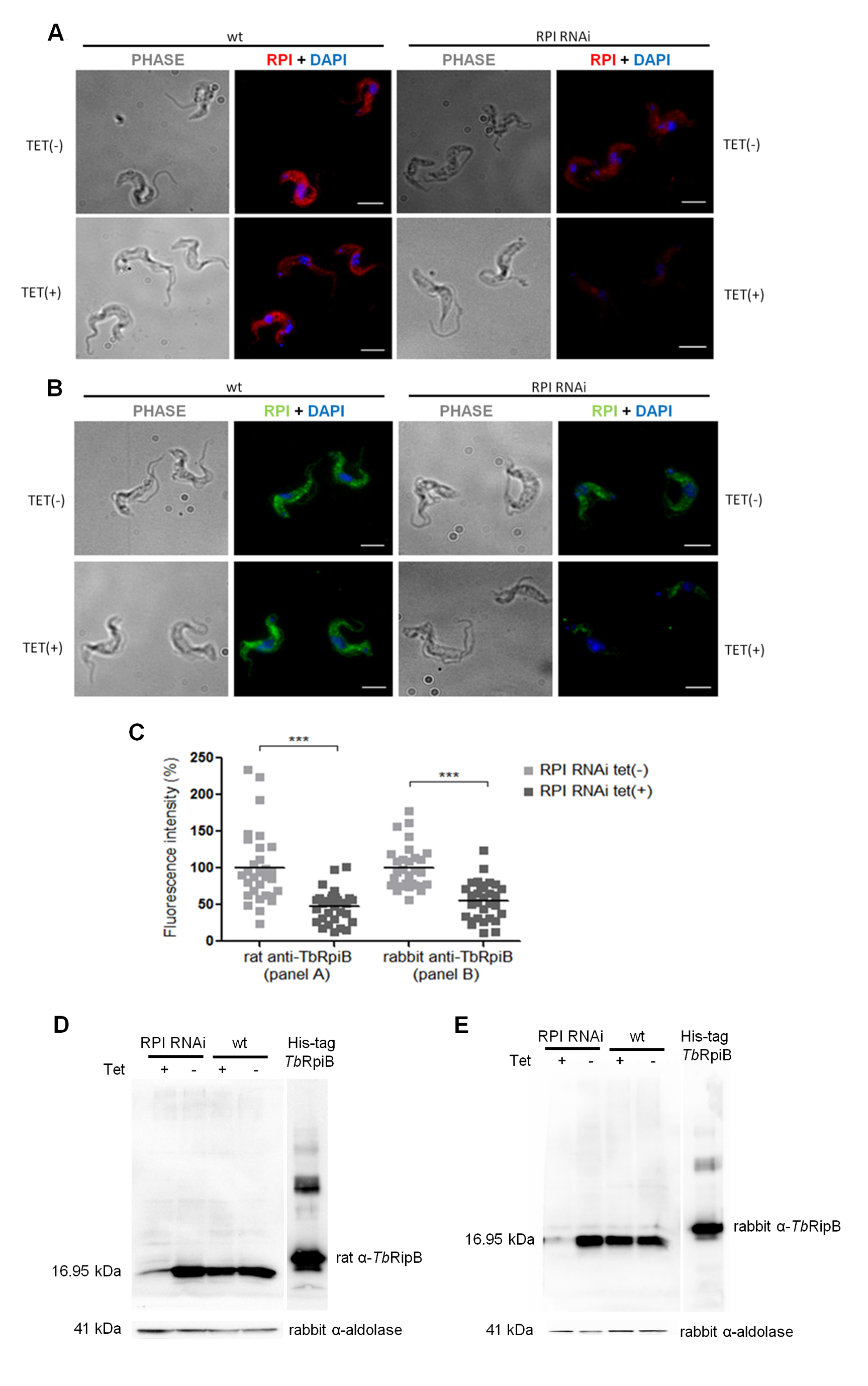

Supplement: S3 Fig — Validation of antibodies against Tb RpiB. Immunofluorescence analysis of T. brucei wt or a representative Rpi RNAi clone in the presence or absence of tetracycline (tet). RNAi induced and uninduced cells were grown for 48 h, then fixed and probed with rat polyclonal anti-TbRpiB (A) or rabbit polyclonal anti-TbRpiB (B) antibody and co-stained with DAPI. Bars, 5 µm. (C) Quantification of TbRpiB fluorescence levels in induced cells [Rpi RNAi tet(+), n = 30] and uninduced cells [Rpi RNAi tet(−), n = 30], using the rat and the rabbit polyclonal anti-TbRpiB antibodies. Data representative of two independent experiments using two different clones. ImageJ software (version 1.43u) was used for fluorescence quantification. p value was calculated by Student's t test (*** p≤0.001, for both p<0.001). (D, E) Whole membrane resulting from Western blot analysis of RpiB levels, in T. brucei wt or a representative Rpi RNAi clone, in the presence or absence of tet. The membrane was probed with rat anti-TbRpiB (1∶100) (D) or rabbit anti-TbRpiB (1∶1000) (E), and after membrane stripping, with rabbit anti-aldolase (1∶5000) for loading control. (TIF) [file pntd.0003430.s003.tif]
